# Beyond Hip Fractures: Other Fragility Fractures’ Associated Mortality, Functional and Economic Importance: A 2-year-Follow-up.

**DOI:** 10.1177/21514593211058969

**Published:** 2021-11-30

**Authors:** Andreas Wiedl, Stefan Förch, Alexander Otto, Leonard Lisitano, Kim Rau, Thilo Nachbaur, Edgar Mayr

**Affiliations:** 1 Abteilung für Unfallchirurgie, Orthopädie, Plastische und Handchirurgie, 39694 Universitätsklinikum Augsburg, Augsburg, Germany

**Keywords:** orthogeriatric, co-management, rib fracture, periprosthetic fracture, periosteosynthetic fracture, ankle joint fracture, pelvic ring fracture, mortality, mobility, activities of daily living

## Abstract

**Background:**

Hip fractures are well researched in orthogeriatric literature. Equivalent investigations for fragility-associated periprosthetic and periosteosynthetic femoral, ankle joint, pelvic ring, and rib fractures are still rare.

The purpose of this study was to evaluate mortality, functional outcome, and socioeconomic parameters associated to the upper-mentioned fragility fractures prospectively in a 2-year follow-up.

**Methods:**

Over the course of a year, all periprosthetic and periosteosynthetic femoral fractures (PPFF), ankle joint fractures (AJ), pelvic ring fractures (PR), and rib fractures (RF), that were treated on a co-managed orthogeriatric ward, were assessed. Parker Mobility Score (PMS), Barthel Index (BI), place of residence, and care level were recorded. After 2 years, patients and/or relatives were contacted by mailed questionnaires or phone calls in order to calculate mortality and reevaluate the mentioned parameters.

**Results:**

Follow-up rate was 77.7%, assessing 87 patients overall. The relative mortality risk was significantly increased for PR (2.9 (95% CI: 1.5–5.4)) and PPFF (3.5 (95% CI: 1.2–5.8)) but not for RF (1.5 (95% CI: 0.4–2.6)) and AJ (2.0 (95% CI: 0.0–4.0)). Every fracture group except AJ showed significantly higher BI on average at follow-up. PMS was, respectively, reduced on average for PR and RF insignificantly, but significantly for PPFF and AJ in comparison to pre-hospital values. 10.0–27.3% (each group) of patients had to leave their homes permanently; care levels were raised in 30.0–61.5% of cases.

**Discussion:**

This investigation provides a perspective for further larger examinations. PR and PPFF correlate with significant increased mortality risk. Patients suffering from PPFF, PR, and RF were able to significantly recover in their activities of daily living. AJ and PPFF conclude in significant reduction of PMS after 2 years.

**Conclusion:**

Any fragility fracture has its impact on mortality, function, and socioeconomic aspects and shall not be underestimated. Despite some fractures not being the most common, they are still present in daily practice.

## Introduction

Hip fractures as the most typical fragility fractures are a common object of investigation in literature. The according examinations confirmed improved outcome in terms of survival and functionality in relation to orthogeriatric co-management.^1-3^ Further relevant fragility fractures like periprosthetic and periosteosynthetic femoral, pelvic ring, ankle joint, and rib fractures are not yet well investigated in this context. Although large-scaled index studies are researched for the respective mortality of the previous mentioned injuries,^4-6^ there are examinations with only few case numbers focusing on other functional and socioeconomic aspects.^7-9^ Furthermore, studies comparing mortality and functional results in between those relevant fragility fractures are still yet to be found in literature.

The aim of the actual investigation was to examine death rates, mobility, activities of daily living, place of residence, and need for care after inward orthogeriatric treatment of these fragility fractures prospectively in a 2-year follow-up.

## Methods

In this 2-year prospective survey, we examined rarer geriatric injuries than hip or vertebral fractures. These are still typical fragility-associated injuries and commonly seen in the daily practice of an orthopedic/geriatric practitioner. The study being a single-center cohort study was conducted at a maximum care hospital. All patients treated on the hospital’s orthogeriatric ward from February 2014 to January 2015 and suffering from periprosthetic or periosteosynthetic femoral (PPFF), ankle joint (AJ), pelvic ring (PR), and rib fractures (RF) were prospectively assessed. A positive approval of the concerning editorial board was on hand (7/11192), as well as the informed consent of patients and/or their legal guardians. Therapy, age, gender, pre-existing Parker Mobility Score (PMS), place of residence (POR), care level, and Barthel Index (BI) on discharge were assessed additionally.

### Therapy

All patients received orthogeriatric co-management by a multi-professional team, including daily ergo- and physiotherapy. Periprosthetic femoral fractures were addressed through osteosynthesis in case of fixed prosthesis. Should the prosthesis have been loosened, treatment was performed through revision arthroplasty. Periosteosynthetic fractures were treated by revision osteosynthesis.

Pelvic ring fractures were mostly addressed conservatively through analgesia and mobilization; unstable Tile-C injuries underwent surgical plate osteosynthesis.

Ankle joint fractures were stabilized by either fibular plate or nail osteosynthesis and depending on injury pattern also screw fixation of the medial malleolus.

Rib fractures underwent conservative treatment, with the occasional use of chest tubes in cases of hemothorax and/or pneumothorax.

Table 1 gives an overview about the respective therapeutical strategies.

**Table 1.** Numbers of Fractures and Respective Therapy.

		Surgical	Conservative
Periprosthetic/periosteosynthetic femoral fractures	N	20	2
	Procedure	Plate osteosynthesis cables revision endoprosthetics medullary nailing	Analgesia and mobilization
Ankle joint fractures	N	N = 16	N = 1
	Procedure	Plate or nail osteosynthesis, screw fixation, and external fixator	Analgesia and mobilization in orthesis
Pelvic ring fractures	N	3	26
	Procedure	Plate osteosynthesis	Analgesia and mobilization
Rib fractures	N	3	16
	Procedure	Chest tube	Respiratory therapy and analgesia

Patients were supported by social workers that helped to provide sufficient care or rehabilitative modalities after discharge. After fractures of the lower extremity and pelvic ring fractures, rehabilitations were organized for these patients if possible.

### Follow-up

Two years upon inward treatment, patients and/or their relatives were contacted through questionnaires about their actual POR, care level, PMS, and BI. In case of no response, a phone call interview was attempted by a maximum of 5 calls. Should the patient have been deceased, the actual date of death was acquired through relatives.

### Statistics

Statistical evaluation was performed through SPPS version 1.0.0.1461 (IBM Corp, Armonk, New York, USA). Linear parameters were examined for their distribution width. Normal distribution was evaluated by Kolmogorov–Smirnov-tests. As no parameter was distributed normally, Kruskal–Wallis tests and chi-square tests were employed to investigate for differences in distribution of continuous and categorical variables. In order to determine significant changes in the parameters PMS and BI over the course of time, paired Wilcoxon tests were performed. 1- and 2-year mortality was calculated fracture-wise, and differences were screened for significance by chi-square tests. The death tables 2013/2015 of Germany’s federal statistical office were used to determine the standardized mortality ratio (SMR). *P*-values were determined as significant at a level of 0.05.

## Results

A total of 87 patients were followed up (77.7% follow-up rate). Table 2 lists respective follow-up rates and age distribution. Kruskal–Wallis test revealed significant different distribution patterns concerning average age in between the fracture groups (*P* = .018). The youngest patients suffered from periprosthetic/periosteosynthetic femoral fractures at an average age of 81.3 years and the oldest accordingly from rib fractures at an average age of 87.1 years.

**Table 2.** Baseline Characteristics.

	N	Age in years	Gender f:m		Follow-up rate	Pre-hospital PMS	BI on discharge
Periprosthetic/periosteosynthetic femoral fractures	22	81.3 (78.0-84.7)	19 : 3	66.7%		6.8 (5.6-7.9)	46.9 (36.5-57.4)
Ankle joint fractures	17	81.9 (78.4-85.4)	15 : 2	89.5%		6.3 (4.9-7.7)	47.5 (38.9-56.2)
Pelvic ring fractures	29	85.6 (83.2-88.1)	24 : 5	76.3%		4.8 (3.8-5.8)	38.4 (31.0-45.8)
Rib fractures	19	87.1 (84.7-89.5)	11 : 8	86.4%		4.2 (2.9-5.5)	42.1 (30.5-53.7)

In general, for all fractures, women were more prevalent than men, but no differences in gender distribution were found in between the fracture groups (*P* = .071).

Fracture-dependent pre-hospital mobility scores were significantly different from each other. Accordingly, patients suffering from rib fractures were the most immobile (*P* = .010). In contrast, regarding BI on discharge, no significant differences in between the fracture groups were revealed (*P* = .275) (Table 2).

### Mortality Rates and Relative Risk

The highest 1- and 2-year mortality was seen in the pelvic ring fracture group (37.9%, a e., 51.7%). Despite the respective absolute fracture-wise 1- and 2-year mortality showing different results in between the fracture groups, no significance was seen (P = .232, a. e. 0.301). Tendentially, the lowest mortality was found after ankle joint fractures (11.8%, a. e. 23.5%). Absolute death rates were intermediate for periprosthetic/periosteosynthetic femoral fractures (31.8%, a. e. 40.9%) and rib fractures (21.1% and 36.8%). Compared to the age-adjusted population, after 2 years, a significant increase of the relative death risk could be detected after periprosthetic/periosteosynthetic femoral fractures (SMR 3.5 95% CI: 1.2–5.8) and after pelvic ring fractures (SMR 2.9 95% CI: 1.5–5.4).

**Figure 1.**
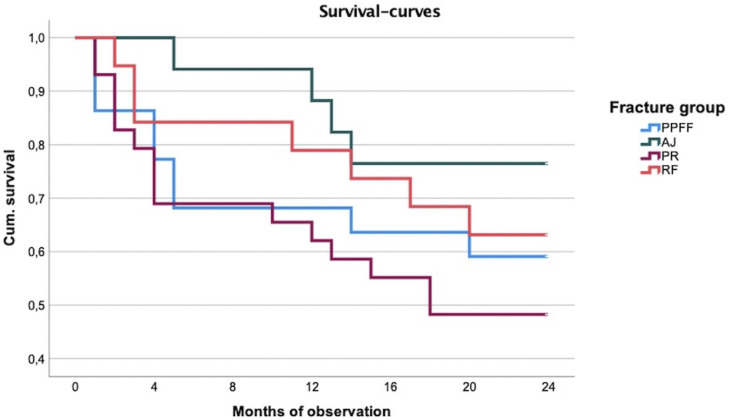
Respective survival curves for each fracture.

The standardized mortality ratio was not significant for ankle joint fractures (SMR 2.0 95% CI: 0.0–4.0) and rib fractures (SMR 1.5 95% CI: 0.4–2.6), the latter being associated to the lowest relative death risk (Table 3 and Figure 1).

**Table 3.** 1- and 2-Year-Mortality Fracture-Wise and According Relative Death Risk.

	1-year mortality	2-year mortality	SMR
Periprosthetic/periosteosynthetic femoral fractures	31.8%	40.9%	3.5 (1.2-5.8)
Ankle joint fractures	11.8%	23.5%	2.0 (0.0-4.0)
Pelvic ring fractures	37.9%	51.7%	2.9 (1.5-5.4)
Rib fractures	21.1%	36.8%	1.5 (0.4-2.6)

SMR = standardized mortality ratio, meaning the relative death risk in comparison to the age-adjusted population.

### Function

Any fracture group experienced an average regain of activities of daily living (ADL) measured by BI. It was the lowest for patients suffering from ankle joint fractures (10.4 (95% CI: −2.0–22.8) and the highest for those suffering from rib fractures (32.5 (95%CI: 20.9–44.1)).

Overall, there was a significant increase in BI from baseline to follow-up for every group except for ankle joint fractures. Patients suffering from periprosthetic/periosteosynthetic femoral fractures reached the highest total BI at follow-up of 78.3 (95% CI: 63.7–93.0). Nevertheless, no significant difference was found comparing activities of daily living at follow-up time in between the groups (*P* = .195).

**Figure 2.**
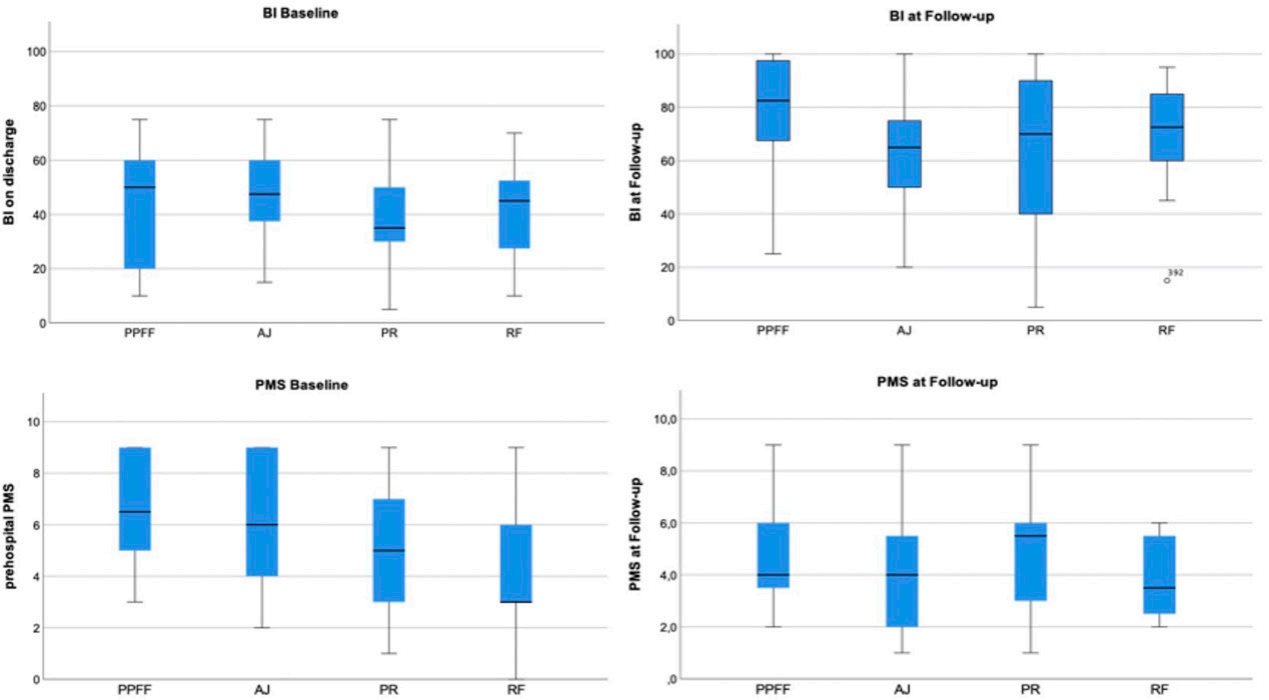
Boxplots for BI and PMS at baseline and follow-up.

Comparing pre-hospital PMS to PMS at follow-up, every fracture entity was associated to an average decline in mobility. Pelvic ring fractures recorded the least change of PMS (−0.4 (95% CI:-1.7–0.9)) and periprosthetic/periosteosynthetic fractures the highest change of PMS (−3.1 (95%CI: −4.6 to −1.7)), the decline only being significant in the latter and ankle joint fracture group. The respective total fracture-dependent Parker Mobility Score at follow-up time showed balanced results without significant differences (*P* = .506) (Table 4, Figure 2).

**Table 4.** BI and PMS at Follow-up and Respective Changes, Statistical Testing Through Wilcoxon-Test.

	BI at follow-up	BI change in comparison to baseline	PMS at follow-up	PMS change in comparison to baseline
Periprosthetic/periosteosynthetic femoral fractures	78.3 (63.7-93.0)	22.5 (1.6-43.4) *P* = .041	4.8 (3.6-6.1)	−3.1 (−4.6 to −1.7) *P* = (.007)
Ankle joint fractures	61.2 (48.2-74.2)	10.4 (−2.0 - 22.8) *P* = .091	4.1 (2.5-5.7)	−2.7 (−5.1 to −0.3) *P* = .049
Pelvic ring fractures	61.8 (44.2-79.4)	27.0 (10.5-43.5) *P* = .011	5.1 (3.5-6.6)	−0.4 (−1.7 - 0.9) *P* = .438
Rib fractures	69.6 (55.0-84.1)	32.5 (20.9-44.1) *P* = .012	3.8 (2.8-4.8)	−1.1 (−2.3 - 0.1) *P* = .068

### Place of Residence and Care Level

Consistently, rare changes were found in the respective POR for previously home-dwelling patients throughout every group, displaying no significant differences in between groups (*P* = .819). After ankle joint and pelvic ring fractures, most patients (respectively 27.3% in both groups) had to leave their homes permanently.

**Figure 3.**
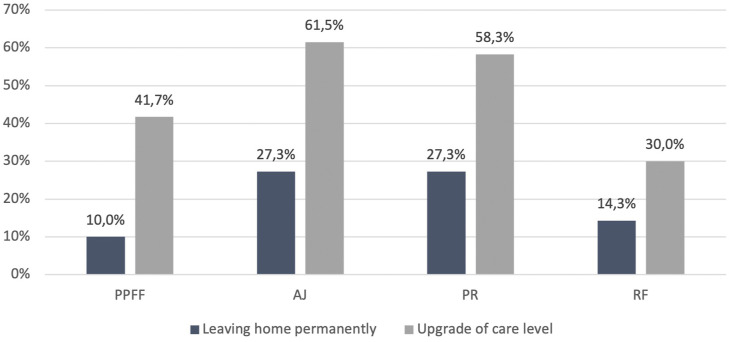
Changes of POR and Care level.

More pronounced alterations were found concerning care level, with an upgrading of the respective care levels in 61.5% of cases of ankle joint fractures and in 58.3% of cases of pelvic ring fractures. While the according rates were slightly lower after periprosthetic/periosteosynthetic fractures (41.7%) or rib fractures (30.0%), in between all groups no significant differences were observed (*P* = .422) (Table 5, Figure 3).

**Table 5.** Changes of POR and Care Level.

	Permanently leaving home	Upgrade of care level
Periprosthetic/periosteosynthetic femoral fractures	10.0%	41.7%
Ankle joint fractures	27.3%	61.5%
Pelvic ring fractures	27.3%	58.3%
Rib fractures	14.3%	30.0%

## Discussion

Most remarkably, we observed a significant increased age-adjusted mortality risk after pelvic ring and periprosthetic/periosteosynthetic femoral fractures. Also, a significant regain of activities of daily living could be observed for periprosthetic/periosteosynthetic femoral, pelvic ring, and rib fractures. Concerning pretraumatic mobility, a significant decline was seen after periprosthetic/periosteosynthetic femoral or ankle joint fractures.

The absolute mortality was the most pronounced for pelvic ring fractures, the lowest for ankle joint fractures, and intermediate for the remaining fracture groups. There were no significant differences in between the respective death rates. In relative terms, high mortality that was seen after pelvic ring fractures in this examination has not been approved by literature yet (1-year mortality in literature: 16.3–22%).^10,11^ 1- and 2-year mortality of periprosthetic/periosteosynthetic femoral and rib fractures is comparable to published results after hip fractures (23.5–28.3% after 1 year^2,12,13^ and 32.5–36.2% after 2 years^12,13^). Nevertheless, after age adjustment, it was revealed that rib fractures are tendentially associated to the lowest and periprosthetic/periosteosynthetic femoral fractures to the highest mortality.

Although patients suffering from pelvic ring fractures experienced a significant recovery of Barthel Index and no substantial change of Parker Mobility Score (being the group with the least change of PMS), the proportion of patients needing more care was 58.3% and of which had to leave their homes permanently was 27.3%. Literature describes similar increases of need for care and changes of accommodation.^14,15^ It must be considered that in this investigation, pelvic ring fractures were mostly treated conservatively and were addressed surgically only in case of unstable fractures according to Tile’s classification. This approach has changed actually in many therapeutical algorithms to this date.^16^

On one hand, patients with ankle joint fractures showed the worst functional outcome with an insignificant regain of Barthel Index, a significant loss of Parker Mobility Score, a proportion of 61.5% who needed more care, and of 27.3% who had to leave their homes permanently. On the other hand, the lowest absolute mortality was observed in this group (11.8% a. e. 23.5%). A possible explanation is an inversion of the survivorship bias. Patients at a low functional level are supposed to be at higher risk for death in the observation period, which would in turn raise the average functional scores at follow-up time. Contrarily for the ankle joint fracture group, many patients with lower-than-average functional scores might have survived. Kadakia et al. and Schray et al. describe a similar 1-year mortality of 6.9%–15.4%, a. e. 10%.^4,9^ Nilsson et al. examined 50 patients suffering from geriatric ankle joint fractures in a 6 months follow-up, the according average age being roundabout 10 years lower than in this investigation. The authors observed every patient being able to return to home.^17^ Schray et al. assessed a comparable cohort of 58 patients treated on an orthogeriatric ward, here again no significant changes of place of residence were observed.^9^ To date, there are no clinical studies investigating on functional outcome including larger samples.

Periprosthetic/periosteosynthetic femoral fractures correlated with the highest age-adjusted mortality risk at an average age of 81.3 years, with the absolute mortality being slightly higher than after hip fractures as mentioned above. Although there was a significant regain of Barthel Index, there was also a significant loss in Parker Mobility Score (being the highest among the fracture groups). In this study, we summarized proximal and distal femoral fractures, which could also have caused biasing effects. In a registry study, Khan et al. determined a 1-year mortality of 21% for 6131 patients, receiving revision arthroplasty after proximal periprosthetic femoral fractures.^5^ Contrarily, a case series including 60 patients suffering from according distal periprosthetic femoral fractures described a lower 1-year mortality of 13%.^18^ This mismatch in between mortality of proximal and distal periprosthetic femoral fractures is approved by another study of Eschbach et al.^8^ Concerning function, Cohen et al. observed a decline of Parker Mobility Score by 2 points and a change of accommodation in 29 cases after 1 year having included 71 patients with periprosthetic femoral fractures.^7^ Even though only every 10th patient in our cohort could not return to home until follow-up, an upgrade of care level was necessary for 41.7%.

Rib fracture group experienced the most pronounced recovery of the activities of daily living and an insignificant loss of mobility. Also, even though not significantly, the least patients were in need for more care and only 14.3% were not able to live at home anymore. Seeing the general context, rib fractures were tendentially (not significantly) associated to the best outcome in our survey. Including patients (>60 years) suffering from rib fractures, Mai et al. described a relative mortality risk of 7.8 for men and of 4.9 for women,^19^ which remarkably exceeds our observation. Long-term analyses concerning functional outcome of rib fractures in the orthogeriatric field could not be found in literature. However, more discharges to nursing homes after acute inward treatment of rib fractures are described, being an indicator of a short-term increase of need for care.^6,20^

Using functional results of orthogeriatric treated hip fractures as a reference, recovery of activities of daily living, changes in mobility, and Parker Mobility Score can also be noticed. One year from discharge, Neuerburg et al. saw a regain of Barthel Index by 19 points and a proportion of 27.5% of patients that were not able to return to home.^1^ Gosch et al. included patients living in nursing homes previously suffering from hip as well as other fragility fractures. One year upon the inward orthogeriatric treatment, the authors observed a decline of Parker Mobility Score (compared to the pre-hospital baseline) by 0.56 respective to 0.63 points.^3^

### Limitations

The group strengths of the respective fracture groups are relatively low, which impairs statistic meaningfulness and might hide significant effects. Overall, 87 patients were assessed completely, and after splitting those into four groups, only small numbers of roundabout 20 patients remained respectively. Literature examines occasionally noticeably bigger cohorts after f. e. pelvic ring^21,22^ (n = 157, a. e. 105), periprosthetic femoral^5^ (n = 6131) a. e.^23^ (n = 106), rib^20^ (n = 277), and ankle joint fractures^4^ (n = 19648). The group strength must be considered in the evaluation of the results’ significance. Our follow-up allowed to assess 1- and 2-year mortality, but other parameters were just captured as a “snapshot” after 2 years, which impedes comparison to other studies employing 1-year follow-ups or other periods. Also, it has to be taken into account that over the years some therapeutical algorithms for the examined injuries have commonly changed, which is mostly the case for pelvic ring fractures. This could also have had a substantial impact on mortality and functional outcome. By summarizing all periprosthetic and periosteosynthetic femoral fractures, a bias could have been induced, for on 1 hand proximal and distal periprosthetic femoral fractures respectively correlating to heterogenous results as mentioned above. On the other hand, periosteosynthetic femoral fractures are not yet well examined and therefore it is not clear whether results are comparable to those of periprosthetic fractures. Nevertheless, our investigation gives conclusions about this not yet thoroughly evaluated field and delivers an impetus for further examinations. To examine more rare fragility fractures for their meaning in the orthogeriatric context, multiple assessment years and group strengths of n>100 should be preferred.

## Conclusion

More rare fragility fractures show comparable results to hip fractures concerning mortality, function, place of residence, and need for care. Being also dependent from fracture severity, significant increased mortality risks were observed for periprosthetic/periosteosynthetic and pelvic ring fractures. Significant recoveries of activities of daily living were seen for all entities except ankle joint fractures, and a significant decline of mobility was found for periprosthetic/periosteosynthetic femoral and ankle joint fractures. The overall best outcome, although not showing significance, was observed after rib fractures.

Other fragility fractures than the most common like hip and vertebral fractures shall not be underestimated. They are of course rarer, but still prevalent throughout.

## Appendix

### Abbreviations

ADL: activities of daily living
AJ: ankle joint fracture
BI: Barthel Index
PMS: Parker Mobility Score
POR: place of residence
PPFF: periprosthetic/periosteosynthetic femoral fracture
PR: pelvic ring fracture
RF: rib fracture
SMR: standardized mortality ratio
